# Induction of *TOC* and *TIC* genes during photomorphogenesis is mediated primarily by cryptochrome 1 in Arabidopsis

**DOI:** 10.1038/s41598-020-76939-w

**Published:** 2020-11-20

**Authors:** Hitoshi Fukazawa, Akari Tada, Lynn G. L. Richardson, Tomohiro Kakizaki, Susumu Uehara, Yasuko Ito-Inaba, Takehito Inaba

**Affiliations:** 1 Department of Agricultural and Environmental Sciences, Faculty of Agriculture, University of Miyazaki, Miyazaki, 889-2192 Japan; 2 AgBioResearch, College of Agriculture and Natural Resources, Michigan State University, East Lansing, MI 48824 USA; 3 Institute of Vegetable and Floriculture Science, NARO, 360 Kusawa, Ano, Tsu, Mie 514-2392 Japan

**Keywords:** Plant sciences, Plant cell biology

## Abstract

The majority of genes encoding photosynthesis-associated proteins in the nucleus are induced by light during photomorphogenesis, allowing plants to establish photoautotrophic growth. Therefore, optimizing the protein import apparatus of plastids, designated as the translocon at the outer and inner envelope membranes of chloroplast (TOC–TIC) complex, upon light exposure is a prerequisite to the import of abundant nuclear-encoded photosynthesis-associated proteins. However, the mechanism that coordinates the optimization of the TOC–TIC complex with the expression of nuclear-encoded photosynthesis-associated genes remains to be characterized in detail. To address this question, we investigated the mechanism by which plastid protein import is regulated by light during photomorphogenesis in Arabidopsis. We found that the albino *plastid protein import2* (*ppi2*) mutant lacking Toc159 protein import receptors have active photoreceptors, even though the mutant fails to induce the expression of photosynthesis-associated nuclear genes upon light illumination. In contrast, many *TOC* and *TIC* genes are rapidly induced by blue light in both WT and the *ppi2* mutant. We uncovered that this regulation is mediated primarily by cryptochrome 1 (CRY1). Furthermore, deficiency of CRY1 resulted in the decrease of some TOC proteins in vivo. Our results suggest that CRY1 plays key roles in optimizing the content of the TOC–TIC apparatus to accommodate the import of abundant photosynthesis-associated proteins during photomorphogenesis.

## Introduction

Chloroplasts are organelles found in photosynthetic tissues of plants, and are thought to have originated from a cyanobacterium engulfed by a eukaryotic cell^1^. Most of the genes that are encoded by the cyanobacterial ancestor have been transferred to the host nuclear genome during evolution. Therefore, the expression of nuclear genes encoding chloroplast proteins, and the import of those proteins into chloroplasts are indispensable for chloroplast development. Protein import from the cytosol to chloroplasts is primarily mediated by the translocon at the outer and inner envelope membranes of chloroplast (TOC–TIC) complexes^2–5^. In *Arabidopsis thaliana* (Arabidopsis), the core TOC complex includes families of Toc159, Toc33 and Toc75 proteins^2^. Among them, Toc75 constitutes the protein-conducting channel, and Toc159 and Toc34 families possess a GTPase domain and serve as chloroplast precursor protein receptors^2,3,6^. Although the role of each TIC component is still controversial, there is a consensus that Tic20 serves as the pore of the TIC complex^7–10^. As such, TOC–TIC complexes play key roles in delivering nuclear-encoded proteins into chloroplasts.

Genes encoding photosynthesis-associated proteins, designated as photosynthesis-associated nuclear genes (PhANGs), are strongly induced by light^11,12^. This necessitates a close relationship between the status of the chloroplast protein import system and the amount of photosynthesis-associated proteins to be imported upon light illumination. For example, light induction of PhANGs does not occur in the *plastid protein import2* (*ppi2*) mutant of Arabidopsis lacking Toc159, a major protein import receptor of photosynthesis-associated proteins^13^. The interpretation of this phenomenon has been that defective plastids send retrograde signals to suppress the expression of PhANGs, thereby preventing the accumulation of unimported precursor proteins in the cytosol^13–15^. On the other hand, the chromophore of phytochromes, phytochromobilin, is synthesized in plastids^16,17^. It has been well known that phytochromes up-regulate a number of PhANGs upon light exposure^18,19^. Hence, one can also argue that *ppi2* does not possess sufficient functional phytochromes to induce the expression of PhANGs. However, little is known about the mechanism by which plastid protein import is coordinated with the expression of PhANGs.

One possible mechanism that may link plastid protein import and the expression of PhANGs is light-regulated expression of *TOC* and *TIC* genes. Several studies have shown that the expression of *TOC* and *TIC* genes is subject to developmental regulation. Among the *TOC159* family, *TOC159* is highly abundant in photosynthetic green tissues, and less abundant in etiolated tissues and roots^20–23^. This is consistent with the proposal that Toc159 is the major protein import receptor for photosynthesis-associated proteins. Likewise, the expression of *TOC33* and *TOC34* is much higher in light-grown plants than in dark-grown plants^24^. *TIC40* and *TIC110* are more abundant in leaves than in roots^22^, and this is consistent with the observation that leaves contain more Tic110 protein compared to roots^25^. These data suggest that the expression of *TOC* and *TIC* genes are somehow up-regulated in green tissues, thereby optimizing the accumulation of TOC and TIC proteins where photosynthetic activity is highest. However, whether this regulation is due to photoreceptor-mediated events or developmental regulation remains elusive.

In this study, we investigated the mechanism by which the expression of PhANGs is coordinated with the status of the TOC–TIC complex in Arabidopsis. We demonstrate that the *ppi2* mutant possesses active phytochromes and cryptochromes even though the mutant failed to induce the expression of the *LHCB* gene in response to light. In contrast, many *TOC* and *TIC* genes are rapidly induced by blue light in WT and *ppi2-2*, suggesting that *TOC* and *TIC* genes are not controlled by plastid retrograde signals. Furthermore, we also show that light induction of *TOC* and *TIC* transcripts is mediated by cryptochrome 1 (CRY1). Based on these results, we discuss the mechanism by which light signals coordinate the expression of PhANGs with plastid protein transport.

## Results

### Phytochromes and cryptochromes are functional in the *ppi2-2* mutant

Previously, we showed that light induction of PhANGs is impaired in the *ppi2-2* mutant^13^. This reduction was most likely due to the action of retrograde signals derived from defective plastids in the *ppi2* mutant. However, many PhANGs have been shown to be induced by phytochromes^11,12^. Therefore, it is also conceivable that the *ppi2* mutation reduces the import of enzymes involved in phytochromobilin biosynthesis, thereby affecting the amount of active phytochromes. The reduction of active phytochromes, rather than retrograde signals from defective plastids, might result in the reduced expression of PhANGs in the *ppi2-2* mutant.

**Figure 1:**
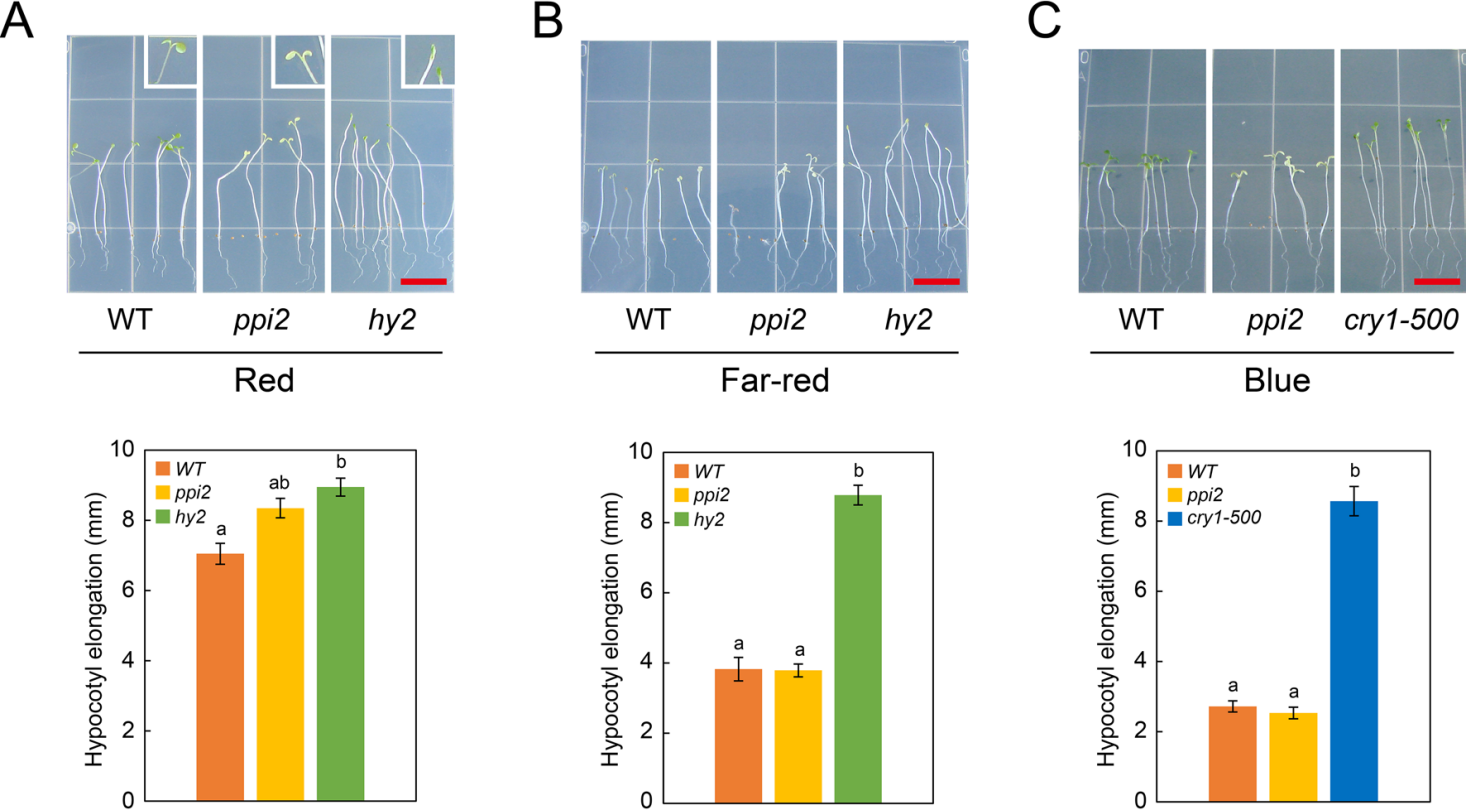
Response of hypocotyl elongation upon monochromatic light irradiation. Plants were grown in the dark for 4 days and then exposed to monochromatic red (**A**), far-red (**B**) or blue (**C**) light for 3 days. Inset in panel (**A**) shows the magnified images of cotyledons in each genotype. Lower panels show quantitative measurement of hypocotyl elongation during 3-day exposure to red (**A**), far-red (**B**) or blue (**C**) light. Error bars indicate the standard error of the mean (*n* ≥ 13). Different letters indicate statistically significant differences between genotypes by Tukey–Kramer multiple comparison test (*P *< 0.05). Bars approximately 1 cm.

To address this question, we investigated whether the *ppi2-2* mutant contains active photoreceptors. Phytochromes have been shown to regulate inhibition of hypocotyl elongation^26^. Specifically, inhibition of hypocotyl elongation by red-light is mediated by phytochrome B (PHYB), whereas phytochrome A (PHYA) mediates the inhibition of hypocotyl elongation by far-red light^27^. Therefore, we exposed WT and *ppi2-2* plants to continuous red or far-red light for 3 days and measured hypocotyl elongation. As a control, we also investigated hypocotyl elongation of the *hy2* mutant that is defective in phytochromobilin biosynthesis^16^. As shown in Fig. 1, hypocotyl elongation of WT was inhibited by both red and far-red light compared to the *hy2* mutant. Far-red light completely inhibited the hypocotyl elongation of the *ppi2-2* mutant, indicating that the amount of PHYA in *ppi2-2* is comparable to that of WT (Fig. 1B). Continuous red-light did not inhibit the hypocotyl elongation of *ppi2-2* completely (Fig. 1A). However, *ppi2-2* as well as WT exhibited cotyledon opening upon continuous red-light illumination (Fig. 1A, inlet). In contrast, *hy2* failed to exhibit cotyledon opening induced by red light. PHYB has been shown to participate in cotyledon opening induced by red light^28,29^. Hypocotyl elongation of *ppi2-2* was comparable to that of WT in the dark (Supplementary Fig. S1). Hence, we conclude that *ppi2-2* mutant contains active PHYB sufficient to induce the red light high irradiance response.

We also investigated whether cryptochromes are functional in *ppi2-2*. CRY1 has been shown to regulate inhibition of hypocotyl elongation by blue light^30^, and cryptochrome 2 (CRY2) has an additive role in this regulation^31^. In contrast to the elongated hypocotyl of *cry1-500* plants, hypocotyl elongation of the *ppi2-2* mutant was completely inhibited by blue light (Fig. 1C). Therefore, we conclude that cryptochromes are also functional in the *ppi2-2* mutant.

Taken together, we concluded that PHYA, PHYB and cryptochromes are functional in the *ppi2-2* mutant. Given the previously observed down-regulation of PhANGs in *ppi2-2*, these results also support the hypothesis that plastid signals play key roles in suppressing the expression of PhANGs upon light exposure in *ppi2-2*.

### Blue light induces the expression of *TOC* and *TIC* genes

**Figure 2:**
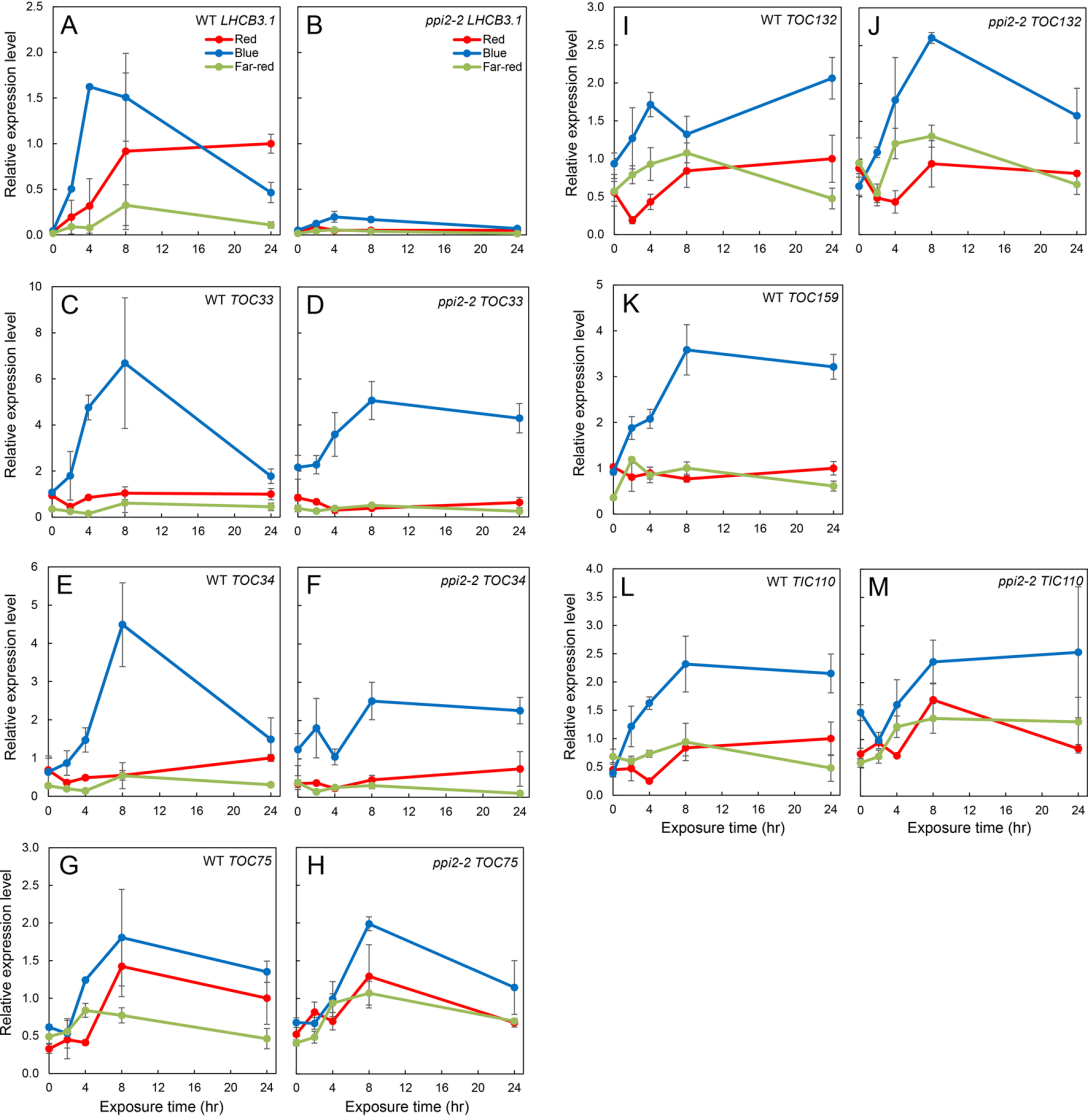
Time course expression analysis of *TOC* and *TIC* genes upon monochromatic light illumination. Plants were grown in the dark for 4 days and then exposed to monochromatic red, far-red or blue light for 0, 2, 4, 8 and 24 h. The mRNA levels were analyzed by real-time PCR and the expression levels were normalized to that of *ACTIN2*. The expression level of each gene in WT after 24-h red light exposure was set to 1. Error bars represent standard error (SE) of the mean (*n* = 3).

A majority of PhANGs are rapidly induced when etiolated plants are exposed to light. Hence, TOC–TIC complexes must mediate the import of a large number of photosynthesis-associated proteins upon light illumination. However, light-dependent regulation of the TOC–TIC complex has not been analyzed in detail. Therefore, we performed a time-course analysis of *TOC* and *TIC* gene expression upon monochromatic light illumination. Both red light and blue light strongly induced the expression of *LHCB3.1* in WT (Fig. 2A). In contrast, none of these light treatments induced the expression of *LHCB3.1* in the *ppi2-2* mutant (Fig. 2B), suggesting that signals derived from *ppi2* plastids suppress light-induced PhANG expression. We next investigated the expression of *TOC* and *TIC* genes. We found that *TOC33*, *TOC34*, *TOC75*, *TOC132*, *TOC159* and *TIC110* genes were induced by blue light in WT (Fig. 2). An intriguing observation was that most of those genes were also induced in the *ppi2-2* mutant in response to blue light (Fig. 2). However, this is in contrast to *LHCB3.1*, which showed a loss of blue-light induction in the *ppi2-2* mutant relative to WT. Furthermore, blue light induction of *TOC132*, a member of the *TOC159* family, was stronger in the *ppi2-2* mutant than in WT. This was most likely due to compensation for the lack of the *TOC159* gene in this mutant.

These data indicate that the expression of *TOC* and *TIC* genes are induced by blue light photoreceptors upon light illumination. Furthermore, this induction is not suppressed by retrograde signals derived from *ppi2* plastids, as is the case for PhANGs.

### Blue light induction of *TOC* and *TIC* genes is regulated by cryptochrome 1

**Figure 3:**
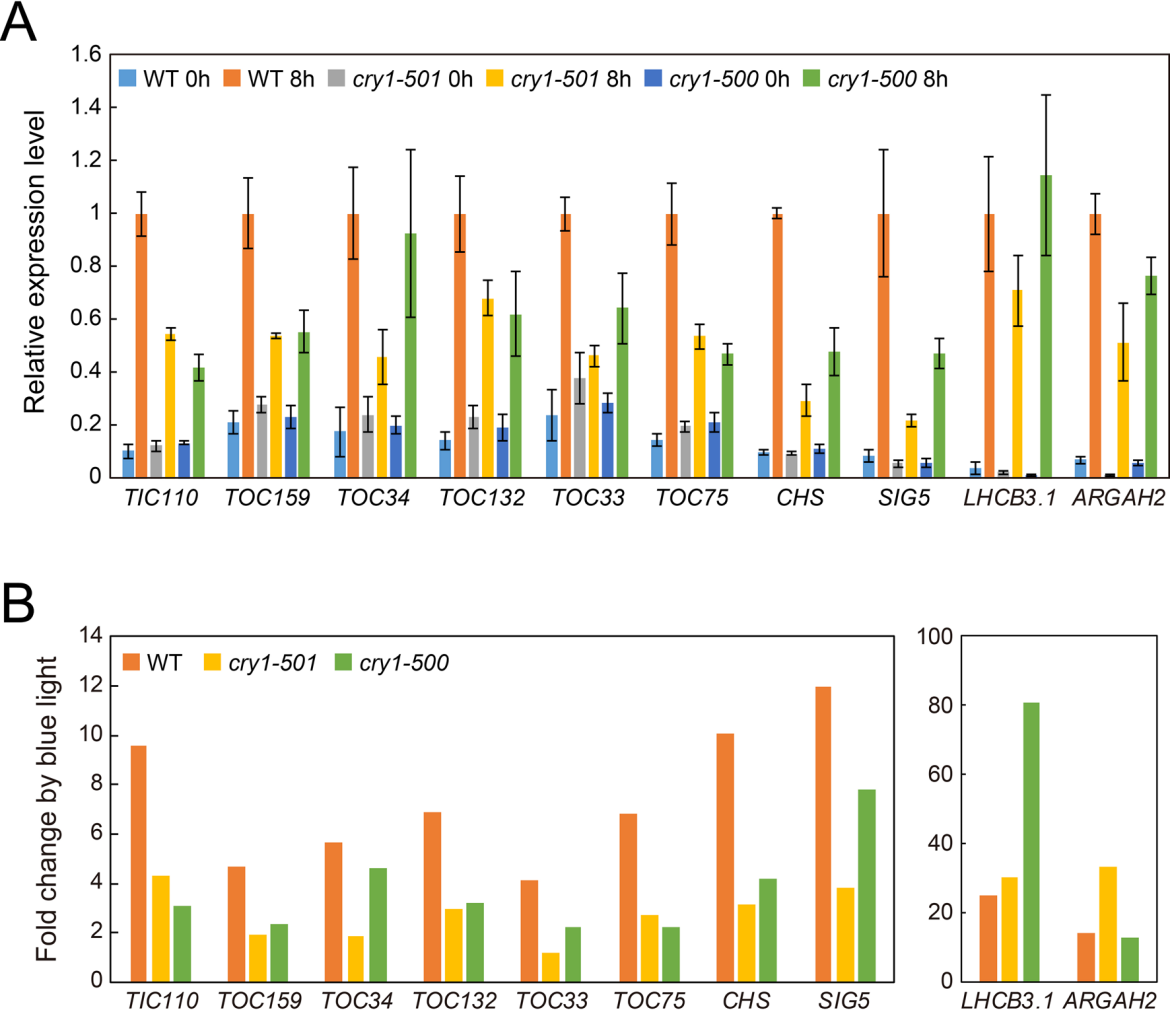
Response of *TOC* and *TIC* genes to blue light in WT and *cry1* mutants. (**A**) Plants were grown in the dark for 4 days and then exposed to monochromatic blue light for 8 h. *CHS* and *SIG5* genes were selected as the control for CRY1 regulated genes, and *ARGAH2* gene was selected as the control for CRY2 regulated gene. The mRNA levels were analyzed by real-time PCR and the expression levels were normalized to that of *ACTIN2*. The expression level of each gene in WT after 8-h blue light exposure was set to 1. Error bars represent standard error (SE) of the mean (*n* = 3). (**B**) Fold change of each gene upon blue light irradiation. The transcript level of each genotype after blue light exposure for 8 h (8 h) was divided by that grown in the dark (0 h).

The fact that blue light induced the expression of *TOC* and *TIC* genes prompted us to further pursue the hypothesis that blue light photoreceptors are responsible for this induction. To this end, we examined whether deficiency of CRY1 affected the blue light induction of *TOC* and *TIC* genes using *cry1* mutants. As positive controls of CRY1 regulated genes, the expression of *CHALCONE SYNTHASE* (*CHS*) and *SIGMA FACTOR5* (*SIG5*) genes was examined^32,33^. We also examined the expression of *ARGININE AMIDOHYDROLASE2* (*ARGAH2*) that is regulated by CRY2^34^. When etiolated WT plants were exposed to blue light for 8 h, all of the *TOC* and *TIC* genes we examined were strongly induced, as were the known CRY1-induced genes, confirming that *TOC* and *TIC* are indeed blue light-induced genes (Fig. 3). The blue light induction of *CHS* and *SIG5* was strongly impaired in *cry1* mutants, as expected (Fig. 3). The *cry1* mutants also failed to induce the expression of *TOC* and *TIC* genes examined, with only the exception of *TOC34* in *cry1-500* mutant (Fig. 3). In contrast, the blue light induction of *LHCB3.1* was virtually unaffected by *cry1* mutations (Fig. 3). Likewise, the fold change of *ARGAH2* by blue light was unaffected or even higher in *cry1* mutants (Fig. 3).

These data indicate that CRY1 primarily mediates the induction of *TOC* and *TIC* genes upon blue light illumination. Because *TOC* and *TIC* genes are weakly induced in *cry1* mutants, we do not exclude the possibility that other photoreceptors, such as CRY2, participate in the blue light induction of *TOC* and *TIC* genes. Nonetheless, the residual induction of *TOC* and *TIC* genes in the *cry1* mutants was comparable to that of the *CHS* and *SIG5* controls.

### Deficiency of cryptochrome 1 affects the accumulation of TOC and TIC proteins

**Figure 4:**
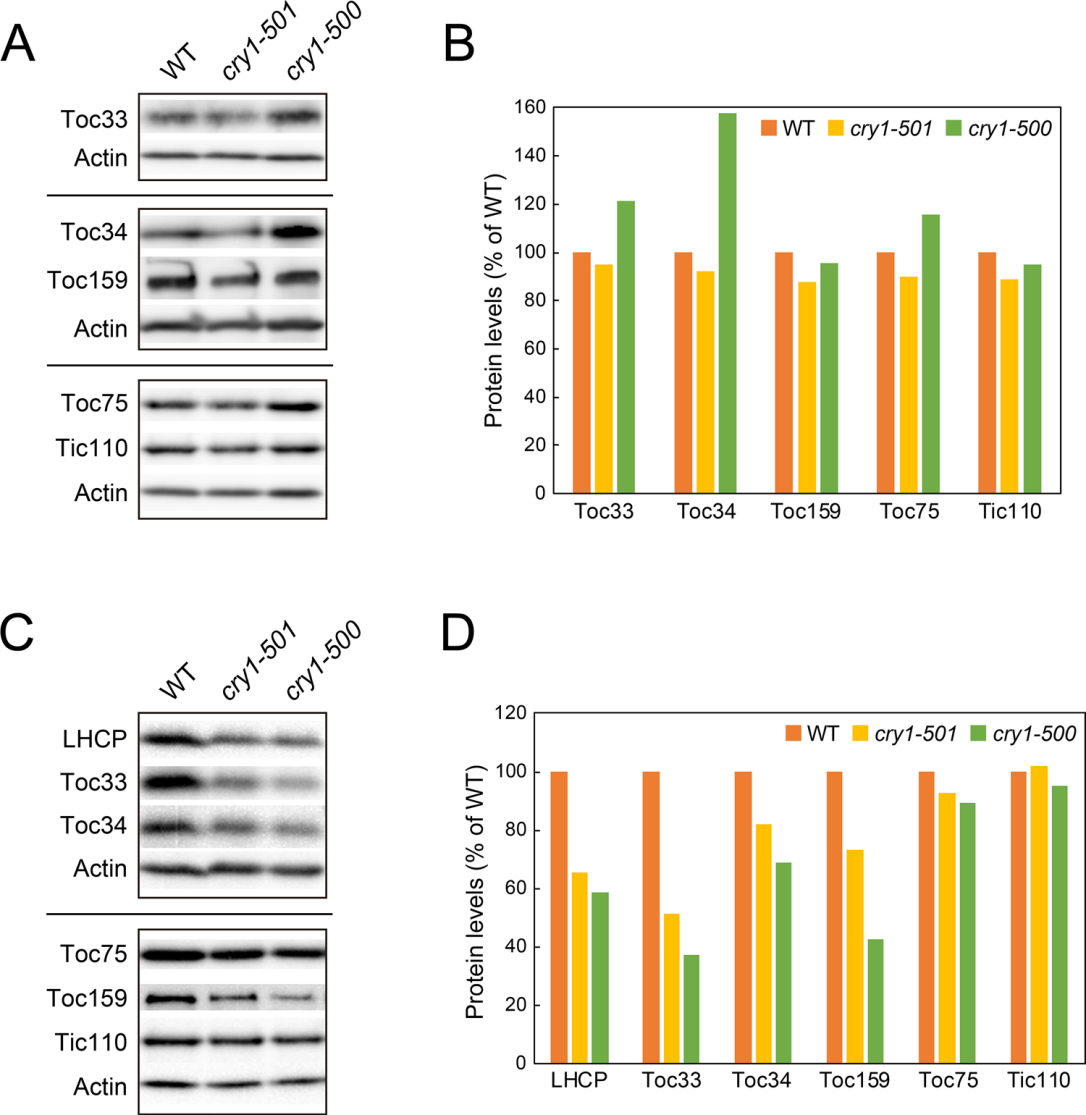
Accumulation of TOC and TIC proteins upon exposure to blue light. Plants were grown in the dark for 4 days (**A**) and then exposed to monochromatic blue light for 3 days (**C**). Extracted proteins were then resolved by SDS-PAGE, and proteins were probed with antibodies indicated at the left. Protein levels in (**A**) and (**C**) were quantified using image acquisition software, normalized to actin levels and shown in (**B**) and (**D**), respectively. The level of each protein in WT was set to 1.

To investigate if a deficiency of CRY1 affects the accumulation of TOC and TIC proteins, we investigated the level of each TOC and TIC protein using immunoblotting. The level of TOC and TIC proteins in the *cry1* mutants was comparable to that in WT in the dark (Fig. 4A,B). When the dark grown WT and *cry1* plants were subsequently exposed to blue light for 3 days, WT accumulated more LHCP, Toc33, Toc34 and Toc159 proteins compared to *cry1* mutants (Fig. 4C). Although the *cry1-500* mutant exhibited a stronger phenotype in terms of protein accumulation, results obtained from two independent alleles were consistent. In contrast, Toc75 and Tic110 proteins were virtually unaffected (Fig. 4C). These conclusions were further supported by quantitative analysis of immunoblot signals (Fig. 4D).

In conclusion, deficiency of CRY1 affects the accumulation of TOC and TIC proteins under blue light. This also supports the hypothesis that blue light induction of *TOC* and *TIC* genes is a physiologically relevant mechanism to regulate the amount of TOC and TIC proteins.

## Discussion

The majority of PhANGs are induced upon light illumination^11,12^. Hence, plastids must optimize the status of the protein import apparatus, designated as the TOC–TIC complex, in response to light to enable the import of abundant photosynthesis-associated proteins. However, the mechanisms that coordinate the constituents of TOC–TIC complexes with the expression of PhANGs remains to be characterized in detail. In this study, we showed that many of the *TOC* and *TIC* genes were induced when etiolated plants were exposed to blue light (Fig. 2). This induction of *TOC* and *TIC* genes was largely mediated by CRY1 (Fig. 3). Furthermore, the prolonged blue light exposure affected the accumulation of some TOC proteins in vivo (Fig. 4). Overall, our results suggest that CRY1 is involved in optimizing the status of TOC–TIC complexes in response to abundant precursors of photosynthesis-associated proteins during photomorphogenesis.

Inhibition of hypocotyl elongation by red and far-red light indicates that the *ppi2* mutant contains active PHYA and PHYB. It has been shown that genes involved in the chlorophyll branch pathway of tetrapyrrole biosynthesis were down-regulated in *ppi*2 mutants^14^. However, *ppi2* seems to produce a sufficient amount of phytochrome holoproteins, as illustrated by inhibition of hypocotyl elongation by red and far-red light (Fig. 1). Likewise, hypocotyl elongation was fully inhibited by blue light in *ppi2* (Fig. 1), indicating that *ppi2* also possesses active cryptochromes. In contrast, *ppi2* failed to induce the expression of *LHCB* through those receptors. These results are consistent with a previous observation that plastid signals can serve as major regulators of light signaling^35,36^. It is also intriguing that, in mutants with damaged plastids, the light response of PhANGs is much more impaired than that of other phytochrome-regulated genes^36^. The nature of signals for that regulation is still obscure. However, previous studies showed that PHYTOCHROME-INTERACTING FACTORs (PIFs) and LONG HYPOCOTYL5 (HY5) were involved in the linkage between light signaling and plastid retrograde signaling^37,38^. Given the fact that both inhibitor treatment and *ppi2* mutation exhibited similar effects on light responsiveness of *LHCB* expression, it is conceivable that common factors are involved in plastid-regulated light responsiveness in both *ppi2* and inhibitor-treated plants.

The induction of *TOC* and *TIC* genes is mediated by CRY1 rather than phytochromes (Figs. 2, 3). This is in contrast to what we know about the regulation of PhANGs. According to transcriptome analysis, a number of PhANGs are regulated by both phytochromes and cryptochromes^19^. Thus, light induction of *TOC* and *TIC* genes appears to be discriminated from that of PhANGs. The reason why Arabidopsis utilizes CRY1 for the induction of *TOC* and *TIC* genes remains obscure. However, it is noteworthy that the induction of *LHCB3.1* by blue light is faster than that by red or far-red light (Fig. 2A). In Arabidopsis, the *SIG1* gene is also induced by both red and blue light. The expression of *SIG1* was strongly induced by red light within 120 min, while blue light induction of *SIG1* expression was much faster^39^. Likewise, the transient induction of *LHCB1.3* in Arabidopsis by blue light pulse was faster than that by red light pulse, while plants treated with red light exhibited higher expression of *LHCB1.3* in later stages (Gao and Kaufman, 1994). Taken together, these data suggest that the expression of *TOC* and *TIC* is rapidly induced by cryptochromes prior to the prolonged induction of PhANGs by phytochromes, allowing timely and efficient transport of photosynthesis-associated proteins. Rapid biogenesis of chloroplasts upon light illumination seems to be, in part, attributable to these mechanisms.

TOC proteins have been shown to be regulated by the ubiquitin proteasome system (UPS)^40–43^. Toc159, Toc75 and Toc33 are polyubiquitinated by a RING-type E3 ubiquitin ligase, and lack of this regulation delays de-etiolation upon light illumination^42^. Given the fact that the level of Toc75 was unaffected even though *TOC75* was reduced in the *cry1* mutants (Fig. 4), it seems that UPS-dependent regulation of Toc75 predominates over its transcriptional regulation during photomorphogenesis. In contrast, CRY1-dependent transcriptional regulation of *TOC159*, *TOC33* and *TOC34* appears to play roles in regulating Toc159, Toc33 and Toc34 protein levels. These data are consistent with the fact that Toc159 and Toc33 families serve as receptor components for precursors^2–4^, and so their levels must be coordinated with light induction of PhANGs. In contrast, Toc75 is involved in the insertion of outer envelope membrane proteins as well as importing chloroplast interior proteins^44^. As such, levels of TOC and TIC proteins are regulated at multiple levels, allowing plants to efficiently import abundant precursor proteins.

In summary, we have uncovered that *TOC* and *TIC* genes, which encode components of the chloroplast protein import apparatus, are induced by blue light through the photoreceptor CRY1. The fact that many photosynthesis-associated proteins, which are the substrates for the TOC–TIC apparatus, also accumulate in response to light illumination suggests that blue light induction of *TOC* and *TIC* genes is a part of a mechanism that coordinates PhANG expression with plastid protein import during photomorphogenesis.

## Methods

### Plant material and growth conditions

All experiments were performed using *Arabidopsis thaliana* Accession Col-0. The *ppi2-2*, *hy2-101* (a kind gift from Prof. Takayuki Kohchi) and *cry1-500* (SALK_042397C; designated as *cry1* in a previous report) mutants were described elsewhere^13,14,16,45^. The *cry1-501* (CS303609) and *cry1-500* (SALK_042397C) were obtained from Arabidopsis Biological Resource Center and the homozygous T-DNA insertion line was screened by PCR. The homozygous *ppi2-2* seeds were obtained using the method as described previously^13^. Seeds of *Arabidopsis thaliana* were sterilized with 70% ethanol and 30% bleach solution and then sown on agar plates containing 0.5 × Murashige–Skoog salt and 1% sucrose. To synchronize germination, all seeds were kept at 4 °C for 3 days in the dark.

### Monochromatic light sources

Light-emitting diodes (LEDs) were used as the monochromatic light sources. LEDs used in the experiments were as follows (EYELA, Tokyo): Red, STICK-mR LED (λmax = 660 nm at 30 μmol m^−2^ s^−1^); Far-red, STICK-mFR (λmax = 735 nm at 25 μmol m^−2^ s^−1^); Blue, STICK-mB LED (λmax = 470 nm at 25 μmol m^−2^ s^−1^). Unless specified, those light sources were used for monochromatic light irradiation.

### Measurement of hypocotyl elongation under monochromatic light irradiation

WT, *ppi2*, *hy2* and *cry1* seeds were sown on 0.5 × MS medium containing 1.5% Agar. Prior to dark treatment, seeds on MS plates were irradiated with continuous white light for 8 h at 22 °C. Then, plates were placed vertically and kept in the dark for 4 days at 22 °C. Before monochromatic light treatment, the position of the top of each hypocotyl was marked on plates. Etiolated plants were then irradiated with red light (660 nm), far-red light (735 nm) or blue light (470 nm) at room temperature for 3 days. After the light treatment, hypocotyl elongation during monochromatic light treatment was measured.

### Time course analysis of gene expression under monochromatic light

WT and *ppi2* mutant seeds were sown on 0.5 × MS medium containing 0.5% Agar. Prior to dark treatment, seeds on MS plates were irradiated with continuous white light at 22 °C for 8 h. Then, plates were kept in the dark for 4 days at 22 °C. Some plants were harvested before monochromatic light treatment. The remaining etiolated plants were then irradiated with red light, far-red light or blue light at room temperature for the times indicated in Fig. 2, and aerial tissues were harvested. The harvested plants were immediately frozen in liquid nitrogen and stored at − 80 °C.

For analysis of *TOC* and *TIC* expression in *cry1* mutants (Fig. 3), WT and *cry1* plants were exposed to blue light for 8 h. Other procedures are the same as stated above.

### RNA isolation and real-time PCR analysis

Total RNA was extracted from aerial tissues of wild-type and mutants using RNAiso reagent (Takara). Then, cDNA was synthesized using the PrimeScript reverse transcription (RT) reagent kit (TaKaRa) with random hexamer and oligo(dT) primers. Real-time PCR was performed on a Thermal Cycler Dice Real-Time System TP870 (TaKaRa) using TB Green Premix ExTaq II (TaKaRa) as described previously^14,46^. Primers used for real-time PCR are listed in Supplementary Table S1. The transcript level of each gene was normalized to that of *ACTIN2*.

### Analysis of TOC and TIC proteins in *cry1* mutants

WT and *cry1* seeds were sown on MS medium containing 0.5% Agar. Prior to dark treatment, seeds on MS plates were irradiated with continuous white light at 22 °C for 8 h. Plates were kept in the dark for 4 days at 22 °C. Then etiolated plants were exposed to blue light for 3 days at room temperature. After light exposure, green aerial tissues were harvested and frozen in liquid nitrogen, and stored at − 80 °C. Total protein extracts from Arabidopsis were obtained by directly homogenizing leaves in SDS-PAGE sample buffer, as described previously^25^.

After protein extraction and quantification, the total protein (20 μg or 10 μg) was analyzed by sodium dodecyl sulfate polyacrylamide gel electrophoresis (SDS-PAGE) using 8%, 12% or 5–20% polyacrylamide gel and immunoblotted with the antisera indicated in the figures. The antibodies against Toc33, Toc34 and Toc159 were kind gifts from Prof. Danny J. Schnell^20,47^. Tic110 and Toc75 have been previously described^25,48^. The LHCP antibodies were a kind gift from Prof. Kenneth Cline. Monoclonal antibody against actin was purchased from CHEMICON. Signals were detected using horseradish peroxidase-conjugated secondary antibodies and chemiluminescence reagent. Signals were quantified using image acquisition software (CS Analyzer; ATTO). All the uncropped blots are shown in Supplementary Figs. S2 and S3.

## Supplementary information

Supplementary Information.
