# Supplementary material for: Induction of TOC and TIC genes during photomorphogenesis is mediated primarily by cryptochrome 1 in Arabidopsis
Source: Sci Rep. 2020 Nov 20;10:20255. doi: 10.1038/s41598-020-76939-w (PMC7680107; doi:10.1038/s41598-020-76939-w)
Supplement: Supplementary file 1 — Supplementary Information. [file 41598_2020_76939_MOESM1_ESM.pdf]

Supplementary Information

Induction of *TOC* and *TIC* genes during photomorphogenesis is mediated primarily by cryptochrome 1 in Arabidopsis

Authors

Hitoshi Fukazawa[1,4], Akari Tada[1,4], Lynn G.L. Richardson[2], Tomohiro Kakizaki[3], Susumu Uehara[1], Yasuko Ito-Inaba[1] and Takehito Inaba[1,*]

[1]Department of Agricultural and Environmental Sciences, Faculty of Agriculture, University of Miyazaki, Miyazaki 889-2192, Japan

[2]AgBioResearch, College of Agriculture and Natural Resources, Michigan State University, East Lansing, MI, USA 48824

[3]Institute of Vegetable and Floriculture Science, NARO, 360 Kusawa, Ano, Tsu, Mie 514-2392, Japan

[4]These authors contributed equally to this work.

***Corresponding author**

Takehito Inaba

TEL/FAX: +81-985-58-7899

e-mail: tinaba@cc.miyazaki-u.ac.jp

Supplementary Table S1. List of primers used for real-time PCR analysis

Gene Name	Forward Primer	Reverse Primer
ACTIN2	5’-GCACCCTGTTCTTCTTACCG-3’	5’-AACCCTCGTAGATTGGCACA-3’
TIC110	5’-CTGCTTCCTCCGACCAACTA-3’	5’-CTGAAGTCGCTAGCCTCACC-3
TOC159	5’-ACAATGGAACCAAAGAGGAGGAG-3’	5’-GGCTTTGCCACATCTACATTCAC-3’
TOC34	5’-GCCAAACCCAAACGAAAGAG-3’	5’-CTTCGACTTGCTAAACCGGAGT-3’
TOC132	5’-TGAGAAACGGACAGAAGAAGAGG-3’	5’-TGTTCCTTCACCTTCCACAACA-3’
TOC33	5’-GAAGCGTGGATCCCGAACT-3’	5’-AGCGCCTATGATAAGAGGGATG-3’
TOC75	5’-CCGCTGCTTCATTCTTTCTGTT-3’	5’-TTCCATCTCCACCACCACCT-3’
CHS	5’-CGTGTTGAGCGAGTATGGAAAC-3’	5’-TGTTTAGAGAGGAACGCTGTGC-3’
SIG5	5’-GAGGGCTGCAAAACCGGTTT-3’	5’-AGCGAGCCTGAGAAGAGCAG-3’
LHCB3.1	5’-TGAACATAACCTTTCTTGTTCCTC-3’	5’-GAGCATTGTAGATTTAGCTGTGAGA-3’
ARGAH2	5’-TTGGGCAGAGAGGAGTTCCC-3’	5’-ACACGGTTCTGCCCTGTCTC-3’

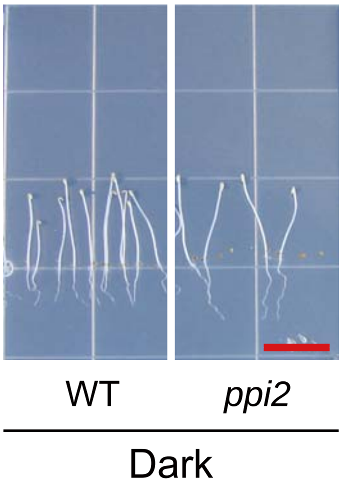

Supplementary Figure S1. Hypocotyl elongation of the dark-grown *ppi2* plants. Plants were grown in the dark for 4 days. Bar = approximately 1 cm.

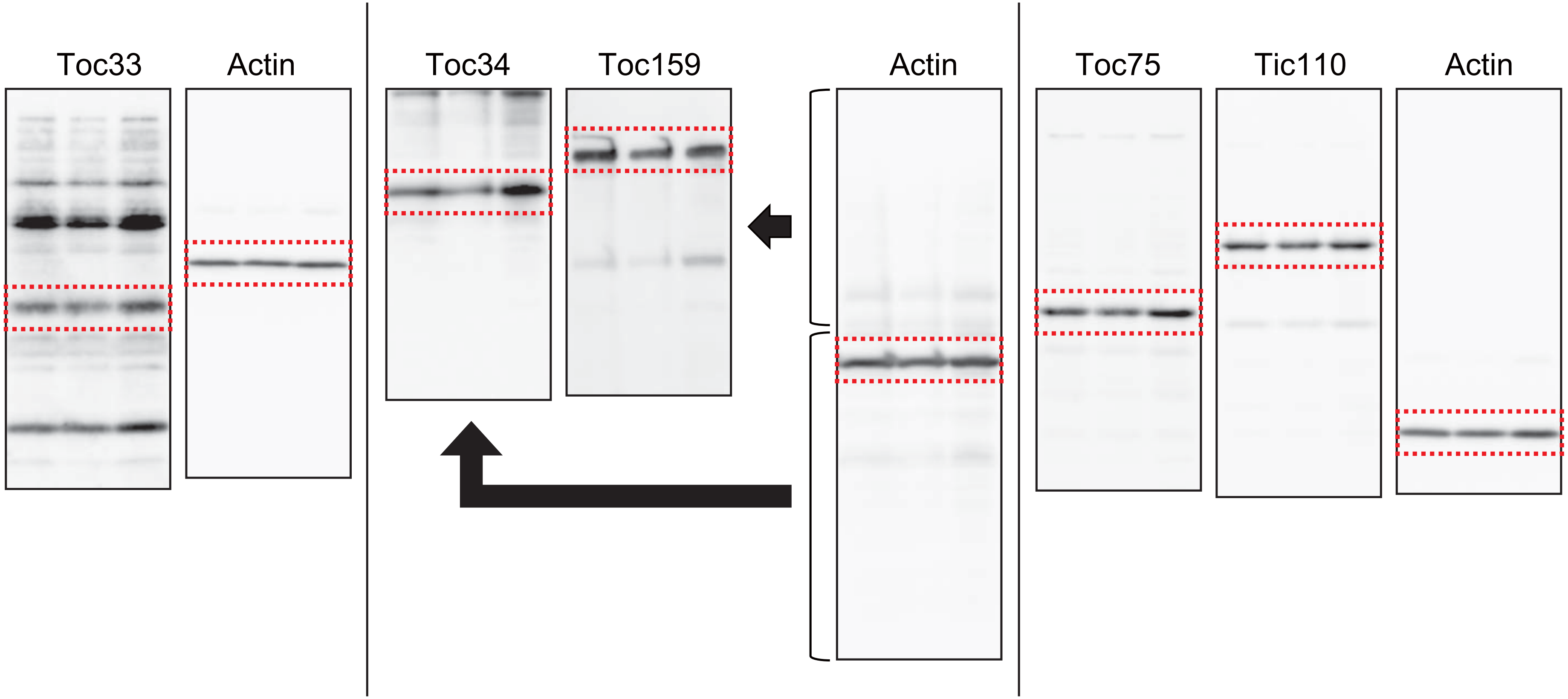

Supplementary Figure S2. Uncropped western blot images for Figure 4A.
Dashed boxes indicate areas that were cropped.

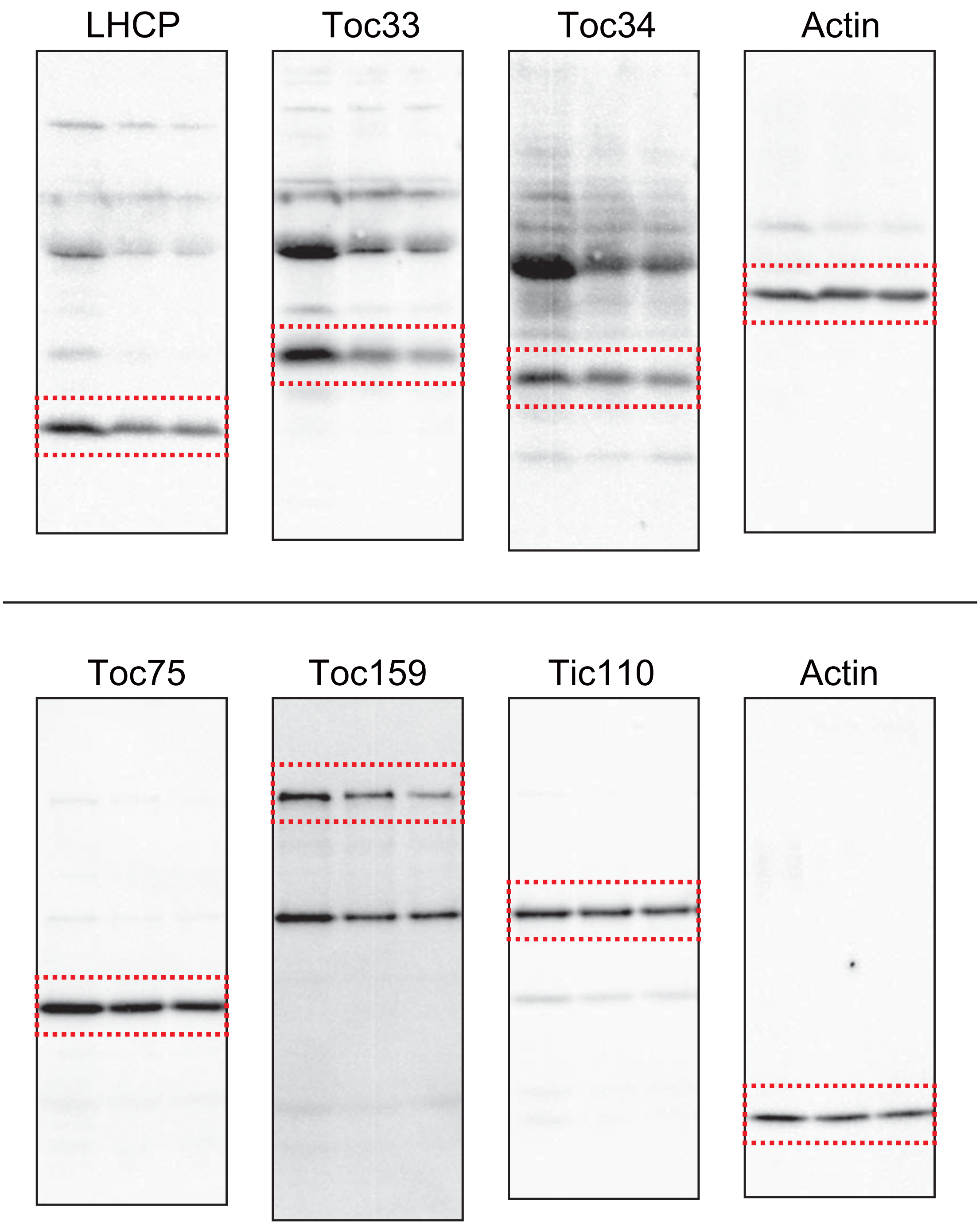

Supplementary Figure S3. Uncropped western blot images for Figure 4C.
Dashed boxes indicate areas that were cropped.
